# Supplementary material for: Content-rich biological network constructed by mining PubMed abstracts
Source: BMC Bioinformatics. 2004 Oct 8;5:147. doi: 10.1186/1471-2105-5-147 (PMC528731; doi:10.1186/1471-2105-5-147)
Supplement: Additional File 2 — The original results of the above study (non-essential files are deleted to keep the file size under the limit set by BMC bioinformatics). [file 1471-2105-5-147-S2.bz2 › chilibotAdditionalFile2/dip05/450ID10477718E10461/html/CCL5_CCR5.html]

 


 **CCL5** and **CCR5** 
  
Found 460 abstracts in PubMed, retrieved 05.  
 

 What does Google say? 
 PDF only 
| .edu only 

---

**Interactive relationship** (e.g. stimulation, inhibition, etc)

**Stimulatory relationship**- Enhanced protein concentrations of RANTES  [ **CCL5** ] , MIP 1a, and MIP 1beta were present in WG lung lesions, indicating redundancy of ligands for  **CCR5**  in affected tissue.  Ref: 12858455 J Rheumatol, 2003
**Neutral relationship**- However, the remaining expression of CCR1 and  **CCR5**  correlated reciprocally with an ongoing unresponsiveness to the CCR1 and  **CCR5**  agonists CCL3 macrophage inflammatory protein 1alpha MIP 1alpha, CCL4 MIP 1beta, and  **CCL5**  regulated on activation, normal T expressed and secreted RANTES  [ **CCL5** ] , all chemokines binding to these two receptors.  Ref: 12885942 J Leukoc Biol, 2003

**Non-interactive relationship** (e.g. studied together, co-existance, homology, etc.)

- METHODS Lung biopsies from 4 patients that had classical features of WG were examined for protein expression of  **CCR5** , RANTES  [ **CCL5** ] , MIP 1a and MIP 1beta using immunohistochemistry.  Ref: 12858455 J Rheumatol, 2003
- After 2 to 4 weeks, L. amazonensis infected mice had significantly delayed and depressed expression of inflammatory cytokines interleukin 12 IL 12, gamma interferon, IL 1alpha, IL 1beta, CC chemokines CC chemokine ligand 3 CCL3 macrophage inflammatory protein 1alpha MIP 1alpha, CCL4 MIP 1beta,  **CCL5**  RANTES  [ **CCL5** ] , MIP 2, and chemokine receptors CCR1, CCR2,  **CCR5**  in foot tissues and draining lymph nodes compared to the expression in L. major infected controls.  Ref: 12874303 Infect Immun, 2003
- **CCR5**  D32 and RANTES  [ **CCL5** ]  28 and 401 polymorphisms were determined by either specific primers or direct sequencing.  Ref: 12858455 J Rheumatol, 2003
